# Association between Childhood Obesity and Metabolic Syndrome: Evidence from a Large Sample of Chinese Children and Adolescents

**DOI:** 10.1371/journal.pone.0047380

**Published:** 2012-10-17

**Authors:** Fangfang Chen, Youfa Wang, Xiaoyi Shan, Hong Cheng, Dongqing Hou, Xiaoyuan Zhao, Tianyou Wang, Di Zhao, Jie Mi

**Affiliations:** 1 Department of Epidemiology, Capital Institute of Pediatrics, Beijing, China; 2 Johns Hopkins, Global Center for Childhood Obesity, Department of International Health, Center for Human Nutrition, Bloomberg School of Public Health, Johns Hopkins University, Baltimore, Maryland, United States of America; 3 College of Pharmacy and Health Science, Drake University, Des Moines, Iowa, United States of America; 4 Department of Pediatric Internal Medicine, the Children's Hospital Affiliated to Capital Institute of Pediatrics, Beijing, China; Aga Khan University, Pakistan

## Abstract

Data about metabolic syndrome (MetS) in children is limited in China. We aimed to assess the prevalence of MetS related components, and their association with obesity. Data were collected as part of a representative study on MetS among 19593 children, aged 6–18 years old in Beijing. General obesity was assessed by body mass index (BMI) and central obesity by waist circumference. Finger capillary blood tests were used to assess triglyceride (TG), total cholesterol (TC) and impaired fasting glucose (IFG). Vein blood samples were collected from a subsample of 3814 children aged 10–18 years to classify MetS. MetS was defined according to the International Diabetes Federation 2007 definition. The associations between MetS related components and the degree and type of obesity were tested using logistic regression models. The prevalence of overweight, obesity, high blood pressure, elevated TG, TC and IFG were13.6%, 5.8%, 8.5%, 8.8%, 1.2% and 2.5%, respectively. Compared with normal weight children, overweight and obese children were more likely to have other MetS related components. In the subsample of 3814 children aged 10–18 years, the prevalence of MetS was much higher in obese subjects than in their normal weight counterparts (27.6% vs. 0.2%). Children with both general and central obesity had the highest prevalence of MetS. Compared with normal weight children, overweight and obese children were more likely to have MetS (overweight: OR = 67.33, 95%CI = 21.32–212.61; obesity: OR = 249.99, 95% CI = 79.51–785.98). Prevalence of MetS related components has reached high level among Beijing children who were overweight or obese. The association between metabolic disorders and obesity was strong.

## Introduction

Childhood obesity has become a worldwide public health concern [1], [2]. Adverse health and psychosocial consequences of childhood obesity are observed in many western populations. They include elevated blood pressure, insulin resistance and dyslipidemia, which are components of metabolic syndrome (MetS). Studies show MetS is strongly associated with increased risk of cardiovascular diseases (CVD) in adults [3], [4]. However, data about MetS in children is rather limited, especially, in developing countries. In addition, approximately 40 criteria are used to assess MetS in children, which make it difficult to consistently identify the disorder and compare results across studies. Although different definitions of MetS are used for children, available data show an increase of MetS in children and adolescents in western populations. Few studies, if any, reported the prevalence of MetS and examine its association with childhood obesity in Asian populations. To provide evidence for intervention and reduce the mortality due to CVD globally, it is critical to document and understand the linkage of childhood obesity to MetS across different populations.

The epidemiology of obesity and related chronic conditions has been changing in the Chinese population, along with China's dramatic economic and nutritional transition. The prevalence of overweight and obesity has increased from approximately 20% in 1992 to 30% in 2002 [5]. Based on the modified international criteria, the prevalence of MetS has reached over 20% in adults [6]. CVDs are now the leading cause of mortality and account for 41% of deaths [7]. National surveys show the prevalence of overweight and obesity among Chinese children increased almost 5 times over a 15-year period (1985–2000) [8].The increase rate is even faster in major cities and coastal areas, compared to inland and rural areas in China. The growing prevalence of obesity in both adults and children indicates enormous burden of chronic diseases in Chinese population, if no effective intervention will take place in the soon future [9].

To better understand the epidemiologic change and provide useful information for intervention, the purpose of the present study was to examine the prevalence of MetS related components and their relationships with obesity among Chinese children and adolescents by using an representative sample of 19 593 children (6–18 years old) from the Beijing Child and Adolescent Metabolic Syndrome study (BCAMS). This study addressed important gaps in current literature by using comprehensive data sources, a large sample covering a wide age range, and objective measures assessed by trained professionals.

## Methods

### Ethics Statement

Written informed consent was obtained from each participant and/or their parents or guardians. The BCAMS protocol study was approved by the Institutional Review Boards and Ethics Committees of Capital Institute of Pediatrics.

### Study Sample

The target population of BCAMS was children under 18 years who lived in Beijing with their parents or guardians at least for 6 months. A stratified , randomly cluster sampling design was used to select subjects from residence communities, kindergartens, elementary schools, middle schools and high schools for different age groups. Four out of eight urban districts and three out of seven rural districts were selected; a total of 35 communities, 13 kindergartens, 22 primary schools and 7 high schools were chosen. Total records of 19 593 children aged 6–18 years who had complete information of physical assessment and finger capillary blood tests were retrieved from BCAMS data files.

Further, the venipuncture blood samples were collected from 3814 children aged 10–18 years who were from the sample described above based on their weight status defined by the Chinese BMI cutoffs [8]: a) 1745 normal weight children b) 1155 overweight children, and c) 914 obese children. We suspect that these venipuncture blood samples might provide better assessment of MetS than the finger capillary blood tests, and thus might reveal stronger association with obesity.

More information about the study design and data collection was provided elsewhere [10], [11].

### Data Collection

Data were collected between April and October in 2004. A survey was delivered to collect background information, family history, and lifestyle information. Physical examinations were conducted and blood samples were collected at selected schools.

#### Anthropometric measurements

Weights and heights were measured twice for each child by the trained staff according to the standardized protocol. Wall-mounted stadiometers were used to measure standing heights without shoes. Heights were measured to the nearest tenth of a centimeter. Beam scales with a maximum weight of 140 kilograms were used to measure weights, which were measured to the nearest tenth of a kilogram while they were wearing light, indoor clothing. Waist circumferences were measured when the children stood comfortably with his or her weight evenly distributed on both feet. The measurement was taken along the midaxillary line, at the midway between the inferior margin of the last rib and the crest of the ilium, in a horizontal plane. Each landmark was palpated and marked, and the midpoint was determined with an inelastic measuring tape measure and marked. The observer sat by the side of the child and fit the tape snugly but not so tightly as to compress underlying soft tissues. The circumference was measured twice for each subject to the nearest 0.1 cm at the end of normal expiration.

#### Fasting blood samples and laboratory tests

Venous blood samples were collected by direct venipuncture after an overnight (minimum 12 h) fasting. The samples were centrifuged, aliquoted and immediately frozen for future analysis of lipids and hormones. Blood samples were analyzed for concentrations of plasma glucose, serum triglycerides (TG), total cholesterol (TC), and high-density lipoprotein cholesterol (HDL-C). Plasma glucose was determined by the glucose oxidase method. Serum TC and TG concentrations were determined using standard enzymatic methods. HDL-C was measured directly. The serum lipid levels and plasma glucose were assayed using the Hitachi 7060C Automatic Biochemistry Analysis System. More related details were provided elsewhere [12].

### Classification of Obesity and Overweight

Body Mass Index (BMI) was calculated for each subject. BMI has been recommended as a reliable and practical measurement, and has been widely used worldwide for obesity screening among adults and children. The International Obesity Task Force (IOTF) reference was used to classify obesity and overweight [13].

*Central obesity* was classified using waist circumference (WC) cut points, in accordance with the national reference in China which has been developed in 2010 [14].

### Classification of MetS and Related Components

IDF international definition, with minor modification mainly related to the WC cut points was used to classify MetS [15]: i) for adolescents aged 10–16, central obesity (WC≥90th percentile) by age and gender according to the Chinese reference for children and adolescents [14], and the presence of any two of the following four factors, ie. elevated BP (systolic ≥130/diastolic ≥85 mmHg), low HDL-C (<1.03 mmol/L), elevated TG (≥1.7 mmol/L ), IFG (fasting plasma glucose ≥5.6 mmol/L); ii) for those aged 16 years or older: the IDF criteria for adults were used, namely central obesity ( WC≥90 cm for Chinese men and ≥80 cm for Chinese women) plus any two of the following conditions: elevated BP (systolic ≥130/diastolic ≥85 mmHg), low HDL-C (<1.03 mmol/L in males and <1.29 mmol/L in females), elevated TG (≥1.7 mmol/L), and IFG (fasting plasma glucose≥5.6 mmol/L).

### Statistical Analysis

Age and gender differences were compared using χ^2^ tests. Logistic regression models were used to examine associations between obesity and MetS components. The analysis were conducted separately for the sample of 19 593 children and the sample of 3 814 children. Potential confounders and covariates including sex and age were adjusted in the analysis.

Adjusted Odds Ratios (ORs) and 95% confidence intervals (CIs) were calculated. Statistical significance was set at *P*<0.05. Data were analyzed with SPSS, version 13.0 (SPSS, Inc., Chicago, Illinois).

## Results

### Prevalence of obesity, and metabolic disorders

As shown in Table 1, the prevalence of overweight and obesity was approximately 20% according to the IOTF reference; the prevalence was much higher in urban than in rural areas, approximately 25% vs 14%. However, overall the urban-rural differences in the prevalence of other MetS related components these metabolic disorders were small though they were statistically significant. In fact, rural participants had higher rates of elevated BP, 18.6% vs. 16.5%.

**Table 1:** Prevalence (%) of obesity and metabolic disorders among Beijing children aged 6–18 years, whole BCAMS sample. population.

	All			Children aged 6–9 yrs			Adolescents aged 10–18 yrs		
	Total	Urban	Rural*	Total	Urban	Rural*	Total	Urban	Rural*
Sample size	19593	9998	9595	5081	2493	2588	14512	7505	7007
Degree of obesity^a^									
Overweight (not obese)	13.6	17.3	9.7	11.2	14.6	8.0	14.4	18.2	10.3
Obese	5.8	7.4	4.1	6.3	8.5	4.3	5.6	7.0	4.1
Elevated blood pressure^b^									
High-normal	9.0	8.4	9.7	8.3	7.1	9.4	9.3	8.8	9.8
Hypertension	8.5	8.1	8.9	10.4	8.8	11.9	7.8	7.8	7.9
Elevated TG^c^	8.8	9.5	8.1	6.0	5.7	6.2	9.8	10.7	8.8
Elevated TC^d^	1.2	1.6	0.8	1.3	1.5	1.0	1.2	1.6	0.7
IFG^e^	2.5	3.1	2.0	1.9	1.6	2.2	2.8	3.6	1.9

Abbreviation: TG, triglyceride; TC, total cholesterol; IFG, impaired fasting glucose. TG, TC and fasting blood glucose were tested based on fasting finger capillary blood.

a Overweight and obesity were diagnosed based on International Obesity Task Force (IOTF) BMI cutoffs.

b Diagnosed by systolic and/or diastolic (K4) blood pressure ≥90th percentile for age and sex from the blood pressure reference standards for Chinese children and adolescents. High-normal was defined as systolic and/or diastolic blood pressure ≥90th–<95th percentile; hypertension ≥95th percentile for age and gender.

c Elevated TG was diagnosed as TG≥1.7 mmol/L.

d Elevated TC was diagnosed as TC≥5.2 mmol/L.

e IFG was diagnosed as glucose ≥5.6 mmol/L.

* χ2-test, all rural and urban differences were significant, *P*<0.01;

### Associations between MetS related components and obesity in the sample of 19593 children aged 6–18 years

Logistic regression models show overweight and obesity were strongly associated with MetS components analyzed (Table 2). The associations (ORs) of obesity with these metabolic disorders almost doubled or tripled those of overweight. For example, compared to normal-weight children, obese and overweight children were 12.84 and 3.83 times more likely to be hypertensive, respectively. Obese and overweight children were also more likely to have elevated TG (ORs 7.07, 2.72 respectively). Those with both general and central obesity were almost 2 to 13 times more likely to have the disorders than the normal-weight children, and had higher risk than having general obesity or central obesity alone.

**Table 2:** Logistic regression model: odds ratios (OR) and 95%CIs of CVD risk factors according to the degree and type of obesity among Beijing children aged 6–18 years, whole BCAMS sample (n = 19593).

	By degree of obesity^a^				By type of obesity^b^					
CVD risk factor	Overweight		Obesity		General obesity only		Central obesity only		General and central obesity	
	*OR*	95% *CI*	*OR*	95% *CI*	*OR*	95% *CI*	*OR*	95% *CI*	*OR*	95% *CI*
Hypertension^c^	3.83	3.38–4.35	12.84	11.15–14.78	4.86	1.36–17.28	2.12	1.37–3.29	13.10	11.36–15.10
Elevated TG^d^	2.72	2.39–3.08	7.07	6.09–8.20	-*	-	2.04	1.43–2.93	7.51	6.45–8.74
Elevated TC^e^	2.61	1.92–3.55	3.94	2.71–5.73	-*	-	1.65	0.60–4.52	4.08	2.80–5.96
IFG^f^	1.22	0.96–1.56	1.93	1.44–2.58	-*	-	0.79	0.29–2.14	1.94	1.45–2.60

Abbreviation: TG, triglyceride; TC, total cholesterol; IFG, impaired fasting glucose. TG, TC and fasting blood glucose were tested based on fasting finger capillary blood.

a Degree of obesity was diagnosed based on International Obesity Task Force (IOTF) BMI cutoffs; Subjects defined as normal weight was used as reference group, and controlled for age and sex.

b Type of obesity was diagnosed simultaneously by IOTF-BMI criteria and the age-,sex-specific waist circumference references for Chinese children and adolescents; Subjects who did not have general obesity or central obesity were used as the reference group.

c Hypertension was defined as systolic and/or diastolic blood pressure ≥95th percentile, the new Chinese reference.

d Elevated TG was defined as TG≥1.7 mmol/L.

e Elevated TC was defined as TC≥5.2 mmol/L.

f IFG was defined as the fasting capillary glucose l≥5.6 mmol/L.

* None had this metabolic disorder.

### The prevalence and associations between MetS and obesity in the subsample of 3814 children aged 10–18 years old who provided venous blood samples

**Figure 1:**
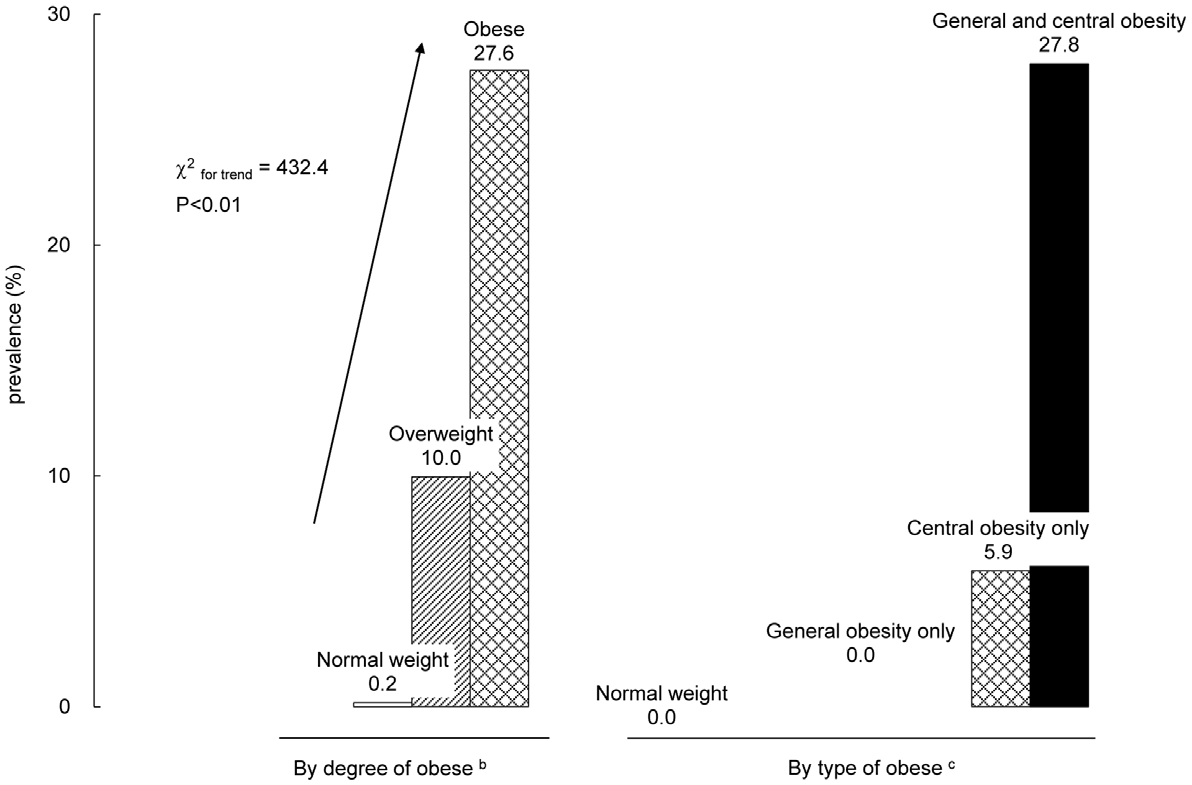
Prevalence (%) of metabolic syndrome by the degree and type of obesity (n = 3814) ^a^. ^a^ Metabolic syndrome was diagnosed by IDF criteria for adolescents; For adolescents aged 10 years to younger than 16 years, MetS was diagnosed with central obesity (WC≥90th percentile) for age and sex by the Chinese reference; and the presence of any two of the following four factors, ie. elevated BP (systolic ≥130/diastolic ≥85 mmHg), low HDL-C (<1.03 mmol/L), elevated TG (≥1.7 mmol/L ), IFG (fasting plasma glucose ^3^ 5.6 mmol/L). For children aged 16 years or older, MetS was diagnosed using IDF criteria for adults, ie: Central obesity (WC≥90 cm for Chinese men and ≥80 cm for Chinese women) plus any two of the following four factors: elevated BP (systolic ≥130/diastolic ≥85 mmHg), low HDL-C (<1.03 mmol/L in males and <1.29 mmol/L in females), elevated TG (≥1.7 mmol/L), IFG (fasting plasma glucose (FPG)≥5.6 mmol/L). ^b^ Degree of obesity was diagnosed based on International Obesity Task Force (IOTF) BMI cutoffs. ^c^ Type of obesity was diagnosed simultaneously by IOTF-BMI criteria and the age-,sex-specific waist circumference 90th percentile by the Chinese reference.

**Figure 1** shows the prevalence of MetS by the degree and type of obesity. There was linear trend of higher prevalence of MetS with the degree of obesity. Only 0.2% of those normal-weight children had MetS, while the prevalence was 10.0% among the overweight and 27.6% among the obese. The prevalence of MetS was more than 100 times higher in obese subjects than in their normal weight counterparts. In addition, those with both general (based on BMI) and central (based on WC) obesity had much higher prevalence of MetS than the other groups.

Overall, the results reported in Tables 3 and 4 were consistent with those for the overall sample aged 6–18 years old, although there were some noticeable differences. The association between metabolic disorders and obesity seemed to be slightly weaker in the subsample. For example, the OR of TG for obesity was 4.43 (3.35–5.85) in this subsample compared to 7.07 (6.09–8.20) in the larger sample with finger capillary blood tests.

**Table 3:** Metabolic disorders among 3814 Beijing children aged 10–18 years by weight status, subsample of BCAMS sample population (n = 3814).*

CVD risk factor	Normal weight (n = 1745)		Overweight (n = 1155)		Obese (n = 914)		*P* value^a^
	Mean	SD	Mean	SD	Mean	SD	
WC (cm)	64.9	5.9	79.8	5.8	91.0	5.9	<0.01
SBP (mmHg)	104.4	10.6	113.3	10.5	119.4	10.7	<0.01
DBP (mmHg)	66.5	8.3	70.9	8.2	74.8	8.3	<0.01
HDL-C (mmol/L)	1.52	0.29	1.30	0.29	1.22	0.30	<0.01
FPG (mmol/L)	5.08	0.78	5.15	0.77	5.17	0.87	<0.01
TG (mmol/L)	0.91	0.52	1.11	0.52	1.29	0.53	<0.01

Abbreviation: WC, waist circumference; SBP, systolic blood pressure; DBP, diastolic blood pressure; HDL-C, high-density lipoprotein cholesterol; FPG, fasting plasma glucose; TG: fasting serum triglyceride.

* Within the whole BCAMS sample, a subsample of 3814 children aged 10–18 years was recruited for venipuncture blood samples. Weight status was defined for the subsample of 3814 children based on the International Obesity Task Force (IOTF) BMI cutoffs; Mean and SD were calculated using covariance analysis adjusted for age and sex.

a *P* values for trend tests across weight status groups adjusted for age and gender.

**Table 4:** Logistic regression models; Odds ratio (OR) and 95%CIs of MetS and MetS component for Beijing adolescents aged 10–18 years according to the degree and type of obesity, subsample* of BCAMS population (n = 3814).

	Degree of obesity^a^				Type of obesity^b^					
	Overweight		Obesity		General obesity only		Central obesity only		General and central obesity	
	*OR*	95% *CI*	*OR*	95% *CI*	*OR*	95% *CI*	*OR*	95% *CI*	*OR*	95% *CI*
**MetS component** c										
Elevated BP	3.36	2.39–4.71	11.07	8.02–15.30	4.06	0.44–37.19	1.59	0.47–5.44	11.53	8.27–16.09
Low HDL-C	3.16	2.47–4.03	5.79	4.49–7.47	2.50	0.29–21.44	1.59	0.71–3.52	5.78	4.44–7.52
Elevated TG	2.60	1.97–3.44	4.43	3.35–5.85	-#	-	3.05	1.32–7.02	4.94	3.69–6.60
IFG	1.16	0.98–1.38	1.62	1.36–1.94	-#	-	0.38	0.15–0.96	1.59	1.33–1.91
**MetS** c	67.33	21.32–212.61	249.99	79.51–785.98	-#	-	-	-	-	-

Abbreviation: HDL-C, high-density lipoprotein cholesterol; IFG, impaired fasting glucose; TG, triglyceride; BP, blood pressure; MetS, metabolic syndrome.

* Within the whole BCAMS sample, a subsample of 3814 children aged 10–18 years was recruited for venipuncture blood samples.

a Degree of obesity was diagnosed based on International Obesity Task Force (IOTF) BMI cutoffs; normal weight subjects were used as reference group; all models adjusted for age and gender.

b Type of obesity was diagnosed simultaneously by IOTF-BMI criteria and the age-,sex-specific waist circumference references for Chinese children and adolescents; subjects who were neither general obesity nor central obesity were used as the reference group; all models adjusted for age and gender.

c MetS and its components were diagnosed according to the IDF criteria for adolescents: For adolescents aged 10∼15, MetS was diagnosed with central obesity (waist circumference ≥90th percentile for age and gender by the Chinese reference; and the presence of any two of the following four factors, ie: elevated BP (systolic ≥130/diastolic ≥85 mmHg), low HDL-C (<1.03 mmol/L), elevated TG (≥1.7 mmol/L ), IFG (fasting plasma glucose ≥5.6 mmol/L). For children aged 16 years or older, MetS was diagnosed using IDF criteria for adults, ie: Central obesity (WC≥90 cm for Chinese men and ≥80 cm for Chinese women) plus any two of the following four factors: elevated BP (systolic ≥130/diastolic ≥85 mmHg), low HDL-C (<1.03 mmol/L in males and <1.29 mmol/L in females), elevated TG (≥1.7 mmol/L), IFG (fasting plasma glucose (FPG) ≥5.6 mmol/L).

# None had the MetS component.

## Discussion

We found that 19.4% of these were overweight or obese and 5.8% were obese in our sample aged 6–18 years. The prevalence in urban areas almost doubled that in rural areas. Our study showed high prevalence rates of MetS and its related components among overweight and obese Chinese children. The prevalence of MetS varied considerably by children's weight status: it was 27.6% in obese group and 0.2% in normal weight group based on the IDF criteria. The prevalence of MetS among obese children in our study population was much higher than that in Japanese (17.7%) [16] and was almost the same as reported in the US (27.8%) [17], [18]. Since obesity was strongly associated with MetS in our sample, it was a useful indicator for screening MetS patients in the target population.

Interestingly, our study found there were significant differences in prevalence of MetS components between rural and urban areas. The prevalence of elevated blood pressure in rural areas was higher than that in urban areas, while prevalence of elevated TG, elevated TC and IGF was higher in urban areas than that in rural areas. These results suggested differences in blood pressure, TG, TC and IGF may be due to different diet, lifestyle and body composition between urban and rural areas [19]. In addition, methods used to test these measures may also affect the results. A study showed finger-tip blood samples tend to overestimate TG, TC and IGF than venous blood sample [20].

Nevertheless, the difference in rural and urban areas is worth of further investigation in order to develop appropriate and effective intervention for metabolic disorders.

The prevalence of childhood obesity in Beijing was increasing steadily over the past two decades, which was consistent with the national trend in China [6], [8]. At present, it is close to the level in north Europe and that of the U.S. in the 1990s [21], [22]. The US national data showed that with the increase in obesity prevalence between 1988–94 and 2001–2006, the overall prevalence of MetS among adolescents had been doubled and increased from 4.2% (6.1% in males and 2.1% in females) [17] to 8.6% (10.8% in males and 6.1% in females) [23]. The dramatic increase in obesity in China and many other countries is a result of environment and lifestyle changes in recent decades [6], [24]. These call on attention and effective efforts to fight the growing obesity epidemic and prevent CVD in China.

Our study showed that central obesity was more closely associated with MetS than general obesity (Figure 1), which is expected as central obesity is one of the MetS components. Its associations with the other individual MetS components (studied as continuous or categorical outcomes) seemed to be stronger than those with general obesity (Table 2) except for a few variables. Thus, data from this large sample of Chinese children support that central obesity indicates greater risk than general obesity.

What is worth of noting is that most (81.0%) of the obese children had both general and central obesity. Thus, when it is difficult to measure both WC and BMI, measuring WC will be acceptable to identify pediatric patients at risk of MetS related disorders, and it may be more cost-effective than measuring BMI.

Our findings provided some useful insights to the between-population differences in MetS and related components and their associations with obesity. For example, their prevalence remained lower than that found in various US ethnic groups, but their associations with obesity seem to be stronger. Several studies, mainly conducted in the U.S., have attempted to examine ethnic differences (mainly among differences among whites, blacks, and Hispanics) in the prevalence of MetS, its components and its related risk factors. Their results suggested ethnic differences, although the results were mixed and inconclusive [2]. The recent US 2001–2006 National Health and Nutrition Examination Survey (NHANES) data showed that about half of U.S. adolescents had at least 1 disordered measurement with an overall MetS prevalence of 8.6% (10.8% in males and 6.1% in females). The prevalence varied across ethnic groups: 11.2% in Hispanic, 8.9% in whites, and 4.0% in blacks [23]. White children had lower prevalence of obesity than the other two ethnic groups [22]. The degree of association between obesity and MetS also varied across ethnic groups.

Similar to adults, US black youth had lower total cholesterol and triglycerides and higher HDL-C levels than white children, and Hispanic children had an increased prevalence of high triglycerides. The Bogalusa Heart Study found higher blood pressure levels in black children even without obesity [2]. A number of studies have shown that black and Hispanic children are similar in MetS disorders except that they are more likely to have insulin resistant than white children [2], MetS rates in black youth are lowest among the three ethnic groups [23]. Thus, a recent AHA expert committee concluded that it may be prudent to consider the use of ethnic-specific criteria for MetS. The genetic and environmental factors that may contribute to ethnic differences in insulin resistance and the other components of MetS are still poorly understood.

The concept of MetS in children and its clinical use remain controversial, and more future research is greatly needed [2], [25]. A recent review of attempts to provide a definition in children highlighted the limitations of deriving or adapting definitions from adults and advocated for a novel and specific approach for children [26]. It is also desirable to developing a universal classification of MetS for the pediatric population, especially, for comparisons between populations [15]. Concerns related to the conceptualization of the MetS include a lack of good understanding of the underlying pathophysiology and considerable variation in its manifestation by age, sex, ethnicity, and maturation phases [2]. A recent study using data of 1789 subjects from three cohort studies in the U.S.-the Fels Longitudinal Study, the Muscatine Study, and the Princeton Follow-up Study examined how thresholds of metabolic components during childhood help predict adult MetS and type 2 diabetes (T2D) based on available metabolic components examined in 1789 subjects [2]. The study concluded that the sensitivity and positive predictive values on the basis of childhood measures remained relatively low, but specificity and negative predictive values were consistently higher, especially for T2D. The authors argued that these components, when examined during childhood, may provide a useful screening approach to identify children who are not at risk. However, whether the results could be generalized to other populations is unclear.

A limitation of the present study was its nature of cross-sectional study in which causality can't be determined. However, this study had a number of strengths. First, we included a large and representative sample. Second, we implemented a set of strategies to ensure the high quality of the data. Third, we applied local and international BMI references to assess the prevalence of overweight (include obesity) and one international criterion to classify MetS. This is one of the earliest studies of MetS conducted in Chinese children and adolescents. China has been undergoing rapid social, economic and nutritional transition. Findings observed in this study were especially meaningful for better understanding the influence of nutritional transition on health and developing effective intervention to prevent obesity and related chronic diseases.

In conclusion, our study showed high prevalence rates of overweight and obesity and MetS related components among children in Beijing. And we also found a strong association between obesity and MetS and its related components. Urgent effort should be made in China, in particular, those areas with fast economic development, to combat the growing obesity epidemic and related chronic diseases. Future research is needed to study the biological pathways linking the various MetS related components, their social, environmental and behavioral risk factors, and how they may differ across different populations.
